# A qualitative interview study applying the COM-B model to explore how hospital-based trainers implement antimicrobial stewardship education and training in UK hospital-based care

**DOI:** 10.1186/s12913-023-09559-5

**Published:** 2023-07-19

**Authors:** Rebecca Turner, Jo Hart, Diane Ashiru-Oredope, Lou Atkins, Christopher Eades, Tim Felton, Emily Howlett, Stephen Rice, Laura Shallcross, Fabiana Lorencatto, Lucie Byrne-Davis

**Affiliations:** 1 Division of Psychology and Mental Health, Faculty of Biology, Medicine & Health, the University of Manchester, Manchester, UK; 2 Division of Medical Education, Faculty of Biology, Medicine & Health, the University of Manchester, Manchester, UK; 3 Clinical and Public Health Group, UK Health Security Agency, London, UK; 4 Centre for Behaviour Change, University College London, London, UK; 5 Department of Infectious Diseases, Manchester University NHS Foundation Trust, Manchester, UK; 6 Department of Critical Care Medicine, Wythenshawe Hospital, Manchester University NHS Foundation, Manchester, UK; 7 Vocal, Manchester University NHS Foundation Trust, Manchester, UK; 8 Population Health Sciences Institute, Faculty of Medical Sciences, the University of Newcastle Upon, Tyne, UK; 9 Institute of Health Informatics, University College London, London, UK

**Keywords:** Antimicrobial Stewardship, Health Service Research, Behavioual Science, Education and training, Healthcare professionals, Interview study

## Abstract

**Background:**

Antimicrobial resistance (AMR) is a major global health threat caused by the inappropriate use of antimicrobials in healthcare and other settings. Antimicrobial stewardship (AMS) is a broad multi-component health services intervention that promotes and monitors the judicious use of antimicrobials to preserve their future effectiveness. A main component of AMS is education and training (E&T). However, there are often discrepancies in how such interventions are implemented and delivered in hospital-based care. The aim of this study was to explore the factors influencing the implementation of AMS E&T in UK hospitals.

**Methods:**

Semi-structured interviews were carried out with AMS E&T trainers in UK hospitals. The interview schedule was developed using the Capability, Opportunity, Motivation = Behaviour (COM-B) model. Participants were identified via professional networks and social media. Interviews were analysed using inductive thematic analysis, followed by deductive analysis using the COM-B model as a framework.

**Results:**

A total of 34 participants (26 antimicrobial pharmacists, 3 nurses, 1 advanced clinical practitioner, 2 infectious disease consultants, 1 microbiologist and 1 clinical scientist). responsible for designing, implementing and evaluating AMS E&T in UK hospitals (five from Northern Ireland, four from Wales, two from Scotland and 23 from England) took part in virtual interviews. Key themes were: (1) The organisational context, including system-level barriers to AMS included competing organisational targets (Reflective motivation and physical opportunity) and the impact of the COVID-19 pandemic on activity (Physical opportunity); (2) Healthcare professionals’ roles and the wider multi-disciplinary team, such that AMS roles were defined and addressed poorly in E&T (Social opportunity); and (3) The individual perception of the need for AMS E&T in hospital-based care, manifest in a perceived lack of conviction of the wider threat of AMR and the resulting need for AMS E&T (Reflective motivation).

**Conclusion:**

This study has identified factors influencing implementation of AMS E&T in UK hospitals and further identified where implemented, AMS E&T did not address real-world challenges. Current AMS E&T needs to be optimised to elicit practice change, with recommendations including training and engaging the wider work-force and drawing upon theoretically-informed intervention development frameworks to inform AMS E&T to better target AMS behaviour change.

**Supplementary Information:**

The online version contains supplementary material available at 10.1186/s12913-023-09559-5.

## Background

 Antimicrobial resistance (AMR) is one of the biggest global challenges of our time [1–3]. The injudicious use of antibiotics has contributed to the development of resistance in many micro-organisms capable of causing disease in humans and animals [2, 4]. In the absence of effective antimicrobials, many common infections and clinical procedures (e.g., obstetric care, major surgery and the care of malignant disorders) become significantly more hazardous and challenging to manage. Leading to significant consequences to patients, causing longer hospital stays, increased risk of health complications and mortality in an already overburdened health systems [4].

Primary care accounts for the majority of antibiotic prescribing, but there has been consistent decreases in primary care prescribing [5]. Whereas sub-optimal antibiotic prescribing is particularly common in hospital settings and is a phenomenon that continues to increase globally [2, 6, 7]. Moreover, the situation has deteriorated in recent times as the significant challenges of the COVID-19 pandemic have had a precipitous, negative impact on antimicrobial use [8–10]. Antimicrobial stewardship (AMS) in hospital-based care, involves targetting multiple behaviours around the appropriate use of antimicrobials by HCPs in a bid to tackle the growing global threat of AMR. Complex multi-component AMS interventions aim to target the determinants of behaviours (e.g., beliefs, habits and knowledge) to ultimately change clinical team’s behaviours around antibiotic use. Examples of such interventions include the development of decision aid tools to support prescribers’ decision-making to achieve safe and effective antibiotic treatment for patients [11] and smartphone applications to highlight guidelines for antibiotic prescribing in hospital-based care [12, 13].

There are several components to AMS such as audit and feedback, tracking antibiotic prescribing and regularly reporting antibiotic use and resistance [3]. One core component within AMS interventions is education and training (E&T) [3]. Typically, E&T can include a wide range of activities. Cochrane Effective Practice and Organisation of Care (EPOC) Review Group [14], provides clear definitions of professional interventions, including E&T, as presented in Table 1.

**Table 1:** Definitions of education and training, as defined by Cochrane Effective Practice and Organisation of Care (EPOC) Review Group (1)

Name	Description
Educational games	The use of games as an educational strategy to improve standards of care
Educational materials	Distribution to individuals, or groups, of educational materials to support clinical care, i.e., any intervention in which knowledge is distributed. For example this may be facilitated by the internet, learning critical appraisal skills; skills for electronic retrieval of information, diagnostic formulation; question formulation
Educational meetings	Courses, workshops, conferences or other educational meetings
Educational outreach visits, or academic detailing	Personal visits by a trained person to health workers in their own settings, to provide information with the aim of changing practice
Inter-professional education	Continuing education for health professionals that involves more than one profession in joint, interactive learning

1. EPOC. Effective Practice and Organisation of Care. EPOC resource for review authors 2015

E&T is widely used in healthcare [15] and is often the default intervention to promote HCP behaviour change to implement new advances into standard care [16]. Despite huge investments of around £4 billion in healthcare E&T each year [15], there are often discrepancies in what is delivered and therefore achieved [17]. Often E&T approaches may be too generic (i.e., ungrounded in the specific clinical context in which they are implemented) with decisions about the content, mode of delivery and format not being based upon relevant theory or evidence which influences effectiveness [18] and consequently leads to vital learning objectives not being met [19]. In turn, this may influence the practical effectiveness of the E&T, leading to unpredictable and potentially costly consequences for safe patient care [20, 21]. AMS E&T is no different, with great variability in how these interventions are delivered, implemented and evaluated in hospital-based care, despite enormous translational efforts [22, 23]. These inconsistencies in AMS E&T, can lead to unnecessary and inappropriate antibiotic prescribing, causing longer stays in hospital for patients, increased risk of health complications and mortality and excess healthcare costs, whilst all driving the global health threat of AMR [1, 24].

The use of a behavioural model can be instructive for AMS E&T and help us with understanding the challenges faced when implementing E&T interventions to optimise AMS in healthcare [25]. The Capability, Opportunity, Motivation = Behaviour model (COM-B) [25] is a simple model of behaviour accounting for internal and external influences on behaviour. The COM-B model proposes an individual’s capability (physical such as skills and psychological such as knowledge), opportunity (physical such as resources and social such as social norms) and, motivation (reflective such as beliefs and automatic such as habits) are necessary requirements for any behaviour to occur. The COM-B model has been widely used to explore barriers and enablers to implementation in healthcare and healthcare practice change. Some examples of its application include understanding hand hygiene amongst HCPs [26], exploring barriers and enablers to providing diet and physical activity support to young mothers [27] and understanding HCPs barriers and enablers to providing opportunistic behaviour change interventions during routine medical consultations [28]. Whilst other implementation models exist such as the Normalisation Process Theory [29] and determinant frameworks, which specifically explore the barriers and facilitators that influence implementation such as the Theoretical Domains Framework [30, 31], the COM-B model was selected due the model being comprehensive and flexible enough to analyse any behaviour in any context [32]. Additionally, the COM-B model sits within an intervention development framework, the Behaviour Change Wheel (BCW) [25], which links the COM-B components onto the BCW and helps support the selection of intervention strategies and techniques that are likely to effectively address the identified barriers and facilitators of the proposed behaviour.

Exploring how E&T is implemented into health services, specifically in hospital-based care, using the COM-B model [25] can provide us with an understanding of the challenges that are faced when aiming to educate and train HCPs. This research will not only help us to reduce the implementation evidence-based practice gap in AMS but also translate key findings to other health services of need. The aim of this study was to explore the factors influencing the implementation of AMS E&T in UK hospitals.

## Methods

### Study design

Qualitative semi-structured virtual interviews, the interview schedule was developed using the COM-B model [25], guided by criteria for reporting qualitative research COREQ [33], please see S1. The topic guide was developed by experienced health psychology/behavioural science researchers (RT, JH and LBD). The topic guide was then reviewed by the patient and public involvement group facilitated by Vocal, a non for profit organisation aiming to bring together patients, researchers, scientists, carers and other healthcare professionals to enhance research and healthcare [34].

### Participants and researchers

Participants were HCPs (including pharmacists, doctors and nurses) who, as part of their job role, were responsible for the design, implementation and evaluation of AMS E&T in specific hospitals in the UK.

The research team (authors) and work package team (RT, JH, TF, DAO and LBD) was made up of multi-disciplinary colleagues with expertise in antimicrobial stewardship interventions (DAO, TF, LS and CE), medicine (TF and CE), health psychology and behavioural science (RT, JH, LA, FB and LBD), health economics (SR) and patient and public involvement experts (EH).

### Recruitment

Participants were recruited using convenience sampling. Potential participants were identified through professional networks, publicly available information on the NHS organisations’ websites, adverts on twitter and snowballing. Additionally, an online expression of interest form was created and hosted via Qualtrics (www.Qualtrics.com) for potential participants to add their details to be contacted by the research team. An invitation email, consent form and participant information sheet outlining the purpose of the study, the methods being utilised for the interview, data protection and confidentiality, as well as relevant contact information were sent to potential participants. Willing participants responded to the email and interview dates were organised. Informed consent was gained from participants at the start of each interview and audio-recorded, by asking participants if they had any further questions, reading out the consent form and participants providing their consent for each of the statements on the consent form.

### Data collection

Participants were interviewed via MS Teams and/or Zoom (with audio-recording and video-recording) by RT who had no prior relationship with the interviewees. The interviewer was a female post-doctoral researcher, with a PhD in health psychology and great experience in interviewing healthcare professionals. To the best of our knowledge, nobody else was present (it is not possible to verify this for the participants, as they were in their own environments). The factors influencing the implementation of AMS E&T in UK hospitals were explored via semi-structured interview (S2). The semi-structured interview schedule was developed using the COM-B model [25] (see S2), to facilitate exploration of the broad range of potential individual, socio-cultural and environmental barriers and enablers to delivering AMS E&T in hospitals. Other types of interventions, in addition to E&T such as technology platforms to change AMS behaviours were also explored. Interviews lasted approximately between 45 and 60 minutes. All interviews were transcribed using an online auto-transcription service (www.otter.ai), transcripts were checked for accuracy by RT and anonymised by redacting any personal details and information about places or organisations. Transcripts were uploaded into NVIVO 12 Plus. Field notes were not used and transcripts were not returned to participants for comment. A review of the data was carried out approximately every five interview transcripts, discussions were held with the research team and wider academic expert advisory group to further identify gaps to explore in future interviews. Data saturation *(defined as the point in a research process where no new information is expressed in the data analysis, which indicates to the researchers that data collection can end)* [35] was regularly discussed with the research team to consider if new data was being expressed by participants [36, 37]. Sample size was determined pragmatically by recruitment constraints and in line with data saturation.

### Data analysis

Data were analysed using an inductive style of thematic analysis, an approach to explore events, realities, meanings and experiences that have been formed and not constrained by constructs of a specific theory or those developed by the research team [38, 39]. One coder (RT) initially read through the transcripts, making notes for familiarity. This coder then began coding the transcripts. Once initial codes were made, they were gathered to make initial themes. Themes were then reviewed by the work package study team in terms of their meaning within the data, in relation to the specific themes and key themes were identified. Following this, the COM-B model [25] was used to analyse the data deductively as a framework to explore the influences on the specified constructs. Two coders (RT and FL) then independently reviewed the themes in relation to the constructs of the COM-B model, assigning each theme to the COM-B model it was judged to best represent. The final analysis was presented to all co-authors and discussed with the work package study team.

Analysing the data using both inductive and deductive approach allows for a rigorous approach to qualitative analysis [40]. Typically inductive approaches are used to understand what is occurring in the data, without forcing the data into a specific framework and potentially missing factors outside of a specific theory [41]. Following this with a deductive approach then allows the researcher to adopt a more focused and organised method to understanding the influences on implementation using a theoretical framework [42].

### Ethics approval and consent to participate

This study has received ethical approval from the University of Manchester University Proportionate Review Ethics Committee (Reference: 2021-12298-20441).

## Results

### Characteristics of participants

Interviews were conducted with 34 individuals across 34 UK organisations, all of whom were responsible for designing, implementing and/or evaluating AMS E&T in National Health Service (NHS) or Health and Social Care (Northern Ireland) (HSCNI) secondary care hospital services. Among these, 23 (68%) practised in England, five (15%) in Northern Ireland, four (12%) in Wales and two (6%) in Scotland. The professional breakdown of participants was as follows: 26 antimicrobial pharmacists, 3 nurses, 1 advanced clinical practitioner, 2 infectious disease consultants, 1 microbiologist and 1 clinical scientist all of which had a role in AMS E&T.

### AMS education and training

To better understand the context of the types of AMS E&T that participants were designing, implementing and evaluating, a summary is as follows: AMS E&T typically included hybrid workshops delivered face to face and/or virtually as part of trust inductions, specifically targeting junior doctors. Whilst other professional groups such as senior doctors, pharmacists and nurses did receive AMS E&T in some cases, this was very rare and not consistent across hospitals. National resources were drawn upon to develop AMS E&T but the majority of participants developed their own trust specific resources.

### Key themes

Three key themes were identified: (1) Organisational and hospital context; (2) HCP roles and the wider team; and (3) individual perceptions of the need for AMS E&T.

These are described below and presented alongside sub-themes in Table 2.

**Table 2:** Overview of key themes and sub-themes identified as influential on the implementation of AMS education and training in hospital-based care

Key themes	Sub-themes	COM-B domain	Example quote(s)
Organisational and hospital context	Lack of uniform message across the four nations	Social opportunity	*“I think it’s easy if you just have one really clear, strong message. Everyone knows that’s what they should be doing.”* ***P031 (Antimicrobial pharmacist, England)***
	Competing interests within the organisation	Reflective motivation and Physical opportunity	*I know that they have so many other things that are extremely important that they need to consider so it’s, there’s so many things that they have to attend and so many training sessions, that it’s, it maybe isn’t a priority?* ***P022 (Antimicrobial pharmacist, Ireland)***
	Practical barriers	Physical opportunity, Automatic motivation and Psychological capability	*“I think this trust was completely useless at providing resources. So they’ve gotten the learning team, but they’re some IT person who I presume is horrendously overworked, but we’ve messaged multiple times asking different people to support developing e-learning packages and got nothing from nobody really.“* ***P004 (Antimicrobial pharmacist, England)***
	Reduced clinical activity and training due to the impact of COVID-19 on resource	Physical opportunity	*“So social distancing, and the need to access equipment’s and not wanting to overwhelm staff.”* ***P001 (Advanced nurse practitioner, Scotland)***
Healthcare professional roles and the wider multi-disciplinary teams	Belief education and training is not targeting the appropriate determinants of change	Reflective motivation and Social opportunity	*“Then you’ve just taught your junior doctors, but actually, they don’t feel like they can go against what the consultants saying.“* ***P020 (Antimicrobial pharmacist, England)***
	Undefined roles of the wider multi-disciplinary team	Social opportunity	*“We’ve got this whole massive workforce that we’re not engaging with to really drive some useful change.”* ***P011 (Antimicrobial pharmacist, England)***
	Developing professional relationships	Social opportunity	*“I’d say antimicrobial stewardship has got a high profile with our trust. . . It’s just easy to make changes and get support things because everybody knows each other.”* ***P027 (Microbiologist, England)***
Individual perception of the need for antimicrobial stewardship education and training in hospital-based care	Lack of ‘buy in’ in education and training subject area	Reflective motivation and Social opportunity	*“We have an antibiotic management team, you know that it meets quarterly and for the past year, we’ve had no representation from medicine, in our trust. I think that speaks volumes into as to how engaged they are. There’s been no consequences to them not turning up.”* ***P023 (Antimicrobial pharmacist, Ireland)***
	Frustration around the lack of perceived importance of AMR/AMS education and training	Automatic motivation	*“You know, people are tired people and I don’t think people are open to this at the moment.”* ***P003 (Antimicrobial pharmacist, England)***

### Organisational and hospital context

Several system-level barriers were discussed by participants in the implementation of AMS E&T. Key influencers included a lack of uniform messaging across the four nations, competing interests and targets within the healthcare organisation, practical challenges such of the routine rotation of junior doctors and the impact of the COVID-19 pandemic on E&T.

#### Lack of uniform messaging across the four nations (Social Opportunity)

When implementing AMS E&T and developing key resources, there was confusion amongst the participants around developing consistent messages. Often resources were developed in the areas the training developers individually perceived as important, which could be different to what others feel is important. Developing a uniform message across the UK to be used within AMS E&T was suggested as a way to improve the field.

*“It’s very hard for even, you know, experts to work out what the best resources are to use, I’d say. So I think that there is a conflict and understanding what are our main messages”****Participant (P) 024 (Antimicrobial pharmacist, England)***.

*“Again, for me, just wide collaborative approach, but be honest, I don’t know, particularly what works well, and what doesn’t. I think often my teaching is much based on you know, things that I feel are important. But I don’t know if people always feel the same, if that makes sense.”****P002 (Advanced clinical practitioner, England)***.

#### Competing interests within the organisation (Reflective Motivation and Physical opportunity)

Participants described various competing interests and targets within their healthcare organisation. This competition made the prioritisation of AMS E&T challenging. Established AMS E&T programmes were typically described as reactive to help address a current issue or had been a part of their mandatory E&T portfolio for a long period of time.

*“I think the trust have said that there’s so much mandatory training, and we struggled to deliver on that, yeah this isn’t much of a priority as issues such as fire safety and manual handling, though, obviously, in our eyes it is.”****P029 (Antimicrobial pharmacist, England)***

*“I think, you know, the barriers probably is that it’s something that’s often easy to get pushed down the priority list.”* ***P019 (Antimicrobial pharmacist, Wales)***

*“I think sometimes the training is kind of prompted, prompted by incidents that happen.”* ***P031 (Antimicrobial pharmacist, England)***

#### Practical barriers (Physical opportunity, Automatic motivation and Psychological capability)

Practical barriers, such as the clinical rotation of junior doctors every three-to-four months, was perceived as a barrier in delivering AMS E&T. Participants discussed that when they were able to educate and train junior doctors in AMS practices, they were concerned this information would not be retained due to the junior doctors being over-burdened.

*“I think the difficulty with getting junior doctors involved in this kind of stuff is that they rotate on a monthly basis, they’ve got so much other stuff to do with their training, you know, it’s not at the forefront of their mind.”****P002 (Advanced clinical practitioner, England)***

*“The challenge that we find is that just with high levels of rotation, and then high numbers of staff, it’s quite hard to do traditional didactic education sessions in such a way that is sustainable.”* ***P024 (Antimicrobial pharmacist, England)***

The IT infrastructure within the organisation also created a barrier to the implementation of AMS E&T, with support and requests taking a long time to be actioned. Participants discussed how other competing interests were prioritised, most likely due to the IT team being overwhelmed by requests for support.

*“So now I’ve had to put in a call to the IT team to get a link, you know, to be able to download these resources and that takes five days. . . But you know, it’s just why this is so hard?”****P013 (Antimicrobial pharmacist, England)***

*“The other problem is that we have what are called single client computers. So none of those have either a video or microphone. So you have to go and find a laptop to be able to do it. So we’re not really set up properly for virtual.”* ***P018 (Antimicrobial pharmacist, Ireland)***

#### Reduced clinical activity and training due to the impact of COVID-19 on resource (Physical opportunity)

Participants discussed how AMS activity was abandoned during the waves in the COVID-19 pandemic and perceived this to have contributed to the increase in antibiotic use during the pandemic.

*“During the, you know, the waves that we had, antimicrobial stewardship activity just went out the window and like other trusts, you could see in wave one, antibiotic use went up massively.”****P015 (Antimicrobial pharmacist, England)***

*“So initially, during the first first peak, the whole team, was pretty much kind of like pulled in service, and we’re doing different things and we didn’t really do any education training.” ****P005 (Antimicrobial Pharmacist, Wales)***

*“I think COVID has really thrown, everything’s sideways. So it was really difficult to maintain the stewardship, especially last year, because they were just prescribing antibiotics, even though the guidance said not to”.****P014 (Antimicrobial pharmacist, England)***

### Healthcare professionals roles and the wider multi-disciplinary team

Several inter-professional barriers were discussed by participants in the implementation of AMS E&T. Key influences included hierarchies on prescribing practices untargeted by AMS E&T, the lack of defined roles in AMS in the wider team, and the need to develop relationships with senior staff within the organisation across different disciplines to help promote AMS E&T.

#### Belief E&T is not targeting the appropriate determinants of change (Reflective motivation and Social opportunity)

AMS E&T was perceived to raise awareness and provide knowledge on AMR and AMS for junior doctors, but it was thought that E&T did not support AMS in a practical sense. It was perceived that the E&T did not provide trainees with the confidence to challenge senior doctors’ prescribing behaviours. Therefore, they were often overruled in relation to antibiotic prescribing:

*“You have to remember, all the time was focused on the juniors, and the issues are with the seniors. . . I mean, the junior is following their consultants and the consultants as well, they’re not helping them to, you know, to learn.”****P006 (Infectious disease consultant, England)***

*“I think it’s probably the senior doctor’s registrar level at that, we probably need to teach more and those are the harder ones to get access to. I think the doctors are on board, they know the guidelines, they use the guidelines, but sometimes they’re overruled.”* ***P019 (Advanced nurse practitioner, Scotland)***

Developing E&T to help support real-life practice such as confidence building and communication skills was suggested to help overcome this barrier.

*“So therefore, by building up their confidence, they’re much more likely to make the decisions we need and often change the consultants practice.”* ***P010 (Antimicrobial pharmacist, England)***

*”Be really great if you could have a session on communication skills or negotiation dealing with difficult people and stuff like that.“* ***P011 (Antimicrobial pharmacist, England)***

Adopting a behavioural approach to developing AMS E&T was thought to be important, but participants felt that they did not possess the relevant skill-set to be able to achieve this.

*“I wouldn’t say its [using behavioural science] not worked. It’s just I would say its [using behavioural science] not as easy. So it, it put me off a little bit. But I do understand that the impact of the behaviour change, you know, reviewing something from a behaviour change perspective.”****P020 (Antimicrobial pharmacist, England)***

#### Undefined roles of the wider multi-disciplinary team (Social opportunity)

The wider roles of the AMS workforce were typically left undefined and unsupported with a fundamental lack of E&T for these professional groups (e.g., consultants, nurses, allied HCPs). Participants felt that other professional groups such as nurses and senior doctors have a role to play in AMS activities:

*“I’ve been banging on about this for years was how do we encourage our nursing staff to just challenge a little bit more, because they’re the ones who are there 24/7.  .. they’ve got really good handle on whether the patient’s getting better or worse, but yet, they never intervene.”****P011 (Antimicrobial pharmacist, England)***

*“I think nurses, I think, you know, within our own trust, I think there’s that there’s a definite gap within their stewardship training for them.”* ***P022 (Antimicrobial pharmacist, Ireland)***

*“I think that the group of doctors that we miss is the middle grades. They’re the hardest group to target and actually, they probably need the training the most.”* ***P027 (Microbiologist, England)***

#### Developing professional relationships (Social opportunity)

Developing relationships with senior staff in different disciplines to help promote the importance of AMR and AMS E&T was often used as a tactic to try and embed AMS E&T into the healthcare system. Senior staff helped to drive change due to their influences and networks within the healthcare organisation.

*“We have relationships with the consultants. . . you do need those on board to kind of really drive change but also we develop mega links with the nurses and the wider MDT as well.”****P002 (Advanced clinical practitioner, England)***

*“So linking in with infection prevention control committee, providing that assurance up to the trust boards, that, you know, we were doing what we were supposed to be doing in terms of NICE (National Institute for Health and Care Excellence) guidance, and, you know, assurances around antimicrobial stewardship, you know, working across the region with colleagues as well to sort of, you know, share good practice.”* ***P011 (Antimicrobial pharmacist, England)***

### Individual perceptions of the need for antimicrobial stewardship E&T in hospital-based care

Several individual barriers were perceived by participants in the implementation of AMS E&T. Key influences included perception of the lack of ‘buy-in’ among stakeholders and frustration around this.

#### Lack of ‘buy-in’ of the E&T subject area (Reflective motivation and Social opportunity)

The subject area of the E&T, in this case AMS, was discussed as influencing whether the E&T was implemented as standard practice or not. AMR was viewed as a debateable area in medicine, with some HCPs not understanding the importance on receiving AMS E&T to improve prescribing practices.

*“Because people just think we pluck things out of thin air, and just, you know, this is just, we’ve just been awkward, so it’s really to try and get them on board and understand the problem, which with AMR is big, and people don’t really see it in front of them.”****P003 (Antimicrobial pharmacist, England)***

*“I still don’t necessarily think non-infectious specialists really get how big the issue actually is now and I think if they knew how big the issue was, now, they would, I think they would probably behave differently.”* ***P016 (Antimicrobial pharmacist, England)***

#### Frustration around the lack of perceived importance of AMR/AMS E&T (Automatic motivation)

The perceived lack of importance of AMR and AMS E&T led to frustration amongst the participants as there appeared to be a lack of support from the organisation to implement such E&T despite evidence of the growing issue of antimicrobial resistance.

*“So about a year ago I got really annoyed and like threw all my toys out of the pram and just said it’s totally not acceptable. You know, if you really believe that, you know what people say that AMR is one of the biggest threats to public health you know, human health and modern medicine, why aren’t we covering it at all in the induction session.”****P011 (Antimicrobial pharmacist, England)***

Burnout of staff during the COVID-19 pandemic was discussed as having an impact upon clinical activity and the provision of AMS E&T, which some participants discussed as challenging due to the importance of AMR to them.

*“So essentially, when they were in height of a pandemic, there wasn’t the burning issue at that point in time. It’s my burning issue. I’m being respectful of what teams were going through, and how overwhelming that was, with what they were dealing with through COVID, staff shortages, all of that compounded the decision to withhold education for a period of time” ****P001 (Advanced nurse practitioner, Scotland)***

## Discussion

The study aimed to explore the factors influencing the implementation of AMS E&T in UK hospitals. The key themes identified organisational level barriers, multi-disciplinary team (MDT) influences and individual barriers and enablers. The COM-B model [25] was drawn upon to help explore and understand the influences on implementation of delivering AMS E&T. Key influences were predominantly driven by physical opportunity, social opportunity, reflective motivation and automatic motivation.

Physical opportunity barriers within the wider organisational and hospital context influenced the implementation of AMS E&T. Establishing E&T within the healthcare organisations portfolio was challenging due to an already crowded E&T programme within hospitals. Developing professional relationships with senior staff in different disciplines to champion AMS E&T, utilising strong and clear messaging, was suggested to help raise awareness of the need for AMS E&T due to their networks and credible position. Other physical opportunity barriers existed such as the routine rotation of junior doctors, organisational IT infrastructure and the influence of the COVID-19 pandemic, which dramatically impacted the provision for E&T and AMS practices. Physical opportunity barriers are unlikely to be targeted via E&T [25], however these barriers can inform future interventions to help work around some of the implementation issues, such as developing and delivering frequent AMS E&T to support junior doctors through their rotations.

Social Opportunity barriers were identified; HCP’s roles and the wider MDT team influenced implementation of AMS E&T. Undefined roles in AMS of the wider team was identified as a key barrier, which has previously been reported across various professions including nurses [43, 44], senior doctors [44] and the wider MDT, including undergraduate trainees [45, 46]. These gaps amongst professional groups within AMS are concerning and are likely to lead to a lack of conviction of the need for AMS E&T within the MDT. The whole MDT need to be engaged, with clear roles and behaviours to support AMS defined.

Reflective motivation barriers about the perceived lack of importance of AMS E&T and the belief AMS E&T is not effective in targeting the appropriate determinant of change were highlighted. Participants described AMS E&T as not tackling real-world challenges. Traditionally healthcare E&T focuses upon the transfer of knowledge from teacher to learner in a didactic fashion [47]. However, knowledge in isolation is a poor mediator of behavioural change as other factors may influence human behaviour [25, 48]. In the area of AMS, the impact of professional hierarchies and power dynamics within MDTs which influence antimicrobial decision-making in hospitals is well researched, but AMS E&T does not address these dynamics at present [1, 24, 49–52]. Thus, whilst AMS E&T has a role in supporting HCPs to confidently recognise sub-optimal or idiosyncratic prescribing, it is unlikely to address the complexities of hierarchical structures within healthcare systems. Such dynamics need to be explored using behavioural and social scientific tools (such as ethnography or phenomenological analysis) to understand how interventions can be developed and adapted to challenge these hierarchies.

Automatic motivation barriers were discussed as participants conversed their frustration with the lack of ‘buy in’ from colleagues and organisations for AMR and therefore AMS E&T. Participants discussed their frustration with the lack of AMS E&T during this time, especially with the precipitous increase of antibiotic prescribing in hospitals [8]. Our data demonstrate the frustrations that emerged in moderating antibiotic use whilst being considerate of staff well-being and inter-personal relationships. There remains real concerns around how HCPs will continue to balance the residual burden of the COVID-19 pandemic against the necessary requirements demanded by AMS interventions [53]. It is likely that such demands represent an area that E&T would be less able to influence with any degree of success; change at the intra- and supra-organisational levels, both in terms of practical infrastructure and culture, would seem necessary to address this situation.

We acknowledge this research has some limitations. Different professional groups were involved in this research, however the differences in these professional groups’ views were not explored. Future research should explore the different experiences of MDTs of the implementation of AMS E&T. A further limitation is due to the use of convenience sampling which perhaps led to the majority of participants being based in England. Significant efforts were made to recruit participants from the devolved nations, but due to the ongoing COVID-19 pandemic and winter pressures in the NHS, this became challenging. The provision of health and social care is devolved from the UK Parliament to local administrations with notable differences in healthcare policy and law between the constituent nations [54]. Thus, we are unable to comment on whether the findings of the study are wholly applicable to all healthcare organisations within the UK, and specifically whether devolved policies play an influence on the implementation of AMS E&T programmes.

### Implications for research

Our interview study highlights gaps, areas for future research in the area of E&T implementation in hospital-based care. Future E&T interventions should identify behaviours and roles for all professional groups and should explore all determinants of behaviour. Further exploring these determinants in more detail, mapping the findings relating to the COM-B model [25] onto the Theoretical Domains Framework [30], would allow a further in-depth understanding of implementation. Then proceeding to using a theoretical intervention development framework such as the Behaviour Change Wheel [25] or Intervention mapping [55] could help education developers consider how to address these some of the barriers utilising theory and evidence. By drawing upon such tools, we can ensure future AMS E&T can be delivered differently, in a way that addresses real-world challenges. However, it is important to note that while AMS E&T can be optimised to address some of these barriers such as challenging beliefs and increasing confidence [56], it is less applicable for tackling infrastructure and resource constraints within healthcare organisations. Interventions and policies that are theory and evidence-based that tackle wider contextual issues are needed to further support appropriate AMS practices.

### Implications for practice

One of the key barriers discussed in this paper is the lack of defined roles healthcare professionals in AMS in practice and therefore in AMS E&T. Behaviour change is unlikely to happen if individuals and teams are not clear on what behaviours they need to deliver and/change. We suggest that formalised competency frameworks such as the WHO health worker AMR competency framework [57] need to be discussed and refined with the MDT to delineate AMS roles clearly drawing upon behavioural specification frameworks to understand what needs to be done, by who, when and where [58]. This needs to be in conjunction with a validated AMS E&T programme, tailored to the local organisational context, linking specifically to what is required from each professional group. Co-developing such resources collaboratively is essential, as top-down, autocratic approaches are less likely to be successful [59].

## Conclusion

This interview study has identified several factors that influenced implementation of AMS E&T in hospital-based care. Wider contextual issues, inter-professional relationships and individual beliefs all play a role in influencing implementation of AMS E&T. When E&T was implemented, it was deemed to have limited impact due to the intervention not addressing real-world challenges such as power dynamics amongst different professional groups. Recommendations for research is for future studies to use a theoretical intervention development frameworks to optimise future AMS E&T, so E&T is delivered differently to target the challenges identified in this work and previous research. Recommendations for policy include educating the wider workforce in AMS practices, ensuring their roles are clearly defined and engaging with key stakeholder such as senior members of staff around the importance of AMR and therefore need for AMS E&T. This will help individuals and teams understand what behaviours they need to deliver and/or change to better tackle AMR.

## Electronic supplementary material

Below is the link to the electronic supplementary material.

Supplementary Material 1

Supplementary Material 2

## Data Availability

The datasets used and/or analysed during the current study are available from the corresponding author on reasonable request.

## Abbreviations

AMR: Antimicrobial resistance
AMS: Antimicrobial Stewardship
COM-B model: The Capability, Opportunity, Motivation=Behaviour model
E&T: Education and training
HCPs: Healthcare professionals
MDT: Multi-disciplinary team
