# Supplementary material for: A qualitative interview study applying the COM-B model to explore how hospital-based trainers implement antimicrobial stewardship education and training in UK hospital-based care
Source: BMC Health Serv Res. 2023 Jul 19;23:770. doi: 10.1186/s12913-023-09559-5 (PMC10357727; doi:10.1186/s12913-023-09559-5)
Supplement: Supplementary file 2 — Supplementary Material 2 [file 12913_2023_9559_MOESM2_ESM.pdf]

A qualitative interview study applying the COM-B model to explore how hospital-based trainers implement antimicrobial stewardship education and training in UK hospital-based care

A review of Antimicrobial Stewardship (AMS) education and training available in secondary care in the NHS

Semi-structured interview schedule

Introduction

Thank you for your time in taking part in this interview. We are interested in your perspective regarding the barriers and facilitators to delivery of Antimicrobial Stewardship (AMS) training. Just to reiterate there is no right or wrong answer and we are simply interested in your opinion.

Questions

Can you provide me a brief summary about the AMS education and training you provide and your current role in AMS education and training?

Probe: type of training delivery, duration, content, follow-up

<u>Instructional methods</u>

Can you tell me about the different instructional methods and components in the AMS education and training you use? *(Psychological capability)*

Can you tell me how deliver these components in the AMS education and training? *(Physical capability)*

Do you always use these different components in the training provision at your trust? *(Psychological capability)*

Probe: Memory

Probe: Habits *(Automatic motivation)*

How confident do you feel delivering the training and all its components? *(Reflective motivation)*

What works in terms of delivery and what do you feel does not work so well? *(Reflective motivation)*

Probe: Emotion (e.g., enjoyment of delivery) *(Automatic motivation)*

How is the training delivery received amongst trainees and or colleagues? *(Social opportunity)*

To what extent do you feel that you have the appropriate resources and the time you require for training delivery? *(Physical opportunity)*

Evaluations

Can you tell me about the evaluations and the data you use to improve AMS education and training programmes? *(Psychological capability)*

How do you carry out AMS education and training evaluations? *(Physical skills)*

How confident do you feel carrying these evaluations out? *(Reflective motivation)*

To what extent do you see this as part of your role? *(Reflective motivation)*

To what extent is this something you always do? *(Automatic motivation)*

Probe: Barriers and facilitators

Do you think there are advantages to evaluating AMS education and training? If so, what are these? *(Reflective motivation)*

Similarity, do you think there are any disadvantages to evaluating AMS education and training? If so, what are these? *(Reflective motivation)*

To what extent do you feel you have the appropriate resources and the time you require for training evaluation? *(Physical opportunity)*

To what extent do you feel you have support from colleagues, trainees, and the organisation to carry out evaluations and use data to improve the AMS education and training? *(Social opportunity)*

Making changes to training provision

Can you tell me about making changes to the training provision? *(Psychological capability)*

Probe: Recommendations for change

How do you make changes to the training provision? *(Physical skills)*

How confident do you feel making potential changes to the training provision? *(Reflective motivation)*

To extent do you see this as part of your role? *(Reflective motivation)*

To what extent is this something you always do? *(Automatic motivation)*

Probe: Barriers and facilitators

Do you think there are advantages to making changes to the AMS education and training? If so, what are these? *(Reflective motivation)*

Similarity, do you think there are any disadvantages to making changes to the AMS education and training? If so, what are these? *(Reflective motivation)*

To what extent do you feel that you have the appropriate resources and the time you require for training improvement? *(Physical opportunity)*

To what extent do you feel you have support from colleagues, trainees, and the organisation to make changes to AMS training provision? *(Social opportunity)*

Final questions

What other interventions or resources are available or delivered at your trust to change AMS behaviours?

Probe: Knowledge based IT systems

What have your overall experiences been of delivering the AMS education and training?

Probe: What works and what does not?

How has the coronavirus pandemic impacted AMS education and training?

Probe: Uptake of training, ability to make changes, delivery and expectations.

How do you imagine AMS education and training evolving in the post COVID era?

Do you think there any barriers or facilitators that we have not discussed that you believe influence the delivery and evaluation of AMS training?
